# Association between *Helicobacter pylori* infection and triglyceride levels: a nested cross-sectional study

**DOI:** 10.3389/fendo.2023.1220347

**Published:** 2023-08-16

**Authors:** Jun Xie, Jinyun Wang, Rong Zeng, Yong Xie

**Affiliations:** ^1^ Department of Gastroenterology, Digestive Disease Hospital, The First Affiliated Hospital of Nanchang University, Nanchang, Jiangxi, China; ^2^ Department of Gastroenterology, The First Affiliated Hospital of Nanchang University, Nanchang, Jiangxi, China; ^3^ Department of Gastroenterology, Jiangxi Clinical Research Center for Gastroenterology, The First Affiliated Hospital of Nanchang University, Nanchang, China

**Keywords:** NHANES, triglycerides, *Helicobacter pylori* infection, females, cdc

## Abstract

**Background:**

Currently, the available evidence regarding the relationship between the lipid profile and Helicobacter pylori (*H. pylori*) infection is limited and conflicting. There is also a dearth of studies that have explored the possibility of sex-specific differences in the association between *H. pylori* infection and triglyceride levels.

**Methods:**

We conducted a cross-sectional study involving 1,146 participants utilizing data from the National Health and Nutrition Examination Survey (NHANES) 1999-2000 conducted in the United States. A logistic regression model was employed to evaluate the association between *H. pylori* seropositivity and triglyceride levels. Subgroup analyses stratified by sex were conducted to explore sex-specific differences in this association.

**Results:**

Serum triglyceride levels were significantly higher in *H. pylori*-seropositive participants than in *H. pylori*-seronegative participants. In the logistic regression analysis, there was a positive correlation between *H. pylori* seropositivity and triglyceride levels (OR=1.231; 95% CI, 1.016-1.491; P=0.033). In the subgroup analysis, the adjusted association between serum triglycerides and *H. pylori* seropositivity was significant in females (OR=1.732; 95% CI, 1.113-2.696; P=0.015) but not in males (OR=1.091; 95% CI, 0.698-1.705; P=0.704).

**Conclusion:**

The association between high triglyceride levels and *H. pylori* infection is specific to the female population.

## Introduction

Helicobacter pylori, a gastric pathogen, has been shown to infect more than half of the global population (1). Since 1994, it has been classified as a Group I carcinogen by the World Health Organization and is capable of causing serious chronic diseases, including chronic gastritis, peptic ulcers, mucosa-associated lymphoid tissue (MALT) lymphoma, and gastric cancer (2, 3). In addition, *H. pylori* infection has been implicated in various extragastric diseases, such as endocrine disorders, osteoporosis, Alzheimer’s disease, and autoimmune thyroid diseases (4–7).

Numerous studies have previously reported a significant correlation between *H. pylori* infection and atherosclerosis, as well as cardiovascular diseases (CVDs) (8–10). Chronic infection with *H. pylori* has been closely associated with alterations in lipid metabolism. Several studies have shown a significant correlation between *H. pylori* infection and triglycerides, total cholesterol (TC), low-density lipoprotein cholesterol (LDL-C), and high-density lipoprotein cholesterol (HDL-C) (8, 11, 12). However, the results are not consistent between different studies. LDL-C was significantly elevated in the subjects with *H. pylori* infection compared to those without *H. pylori* infection. There were no associations among other lipid profiles and *H. pylori* infection. However, another study showed that an *H. pylori*-positive group had lower HDL-C than the *H. pylori*-negative group. Significant differences between the two groups were not found in other lipid profiles, including triglycerides, total cholesterol and LDL-C. Elucidating the precise association between *H. pylori* and cardiovascular risk factors is an important objective in reducing the incidence of CVD.

In this study, we aimed to investigate the potential association between *H. pylori* seroprevalence and lipid profile, utilizing data from the National Health and Nutrition Examination Survey (NHANES) conducted in the United States during 1999-2000. Our primary objective was to shed light on the influence of *H. pylori* infection on cardiovascular risk factors by assessing the relationship between *H. pylori* seroprevalence and the lipid profile.

## Materials and methods

### Study design

All data in this study were obtained from the 1999-2000 NHANES conducted in the United States. NHANES is a research project designed to evaluate the health and nutrition status of both adults and children in the United States and utilizes a complex, multistage, probability sampling design to provide information on the nutrition and health of the general U.S. population (13).

### Inclusion and exclusion criteria for study participants

**Figure 1:**
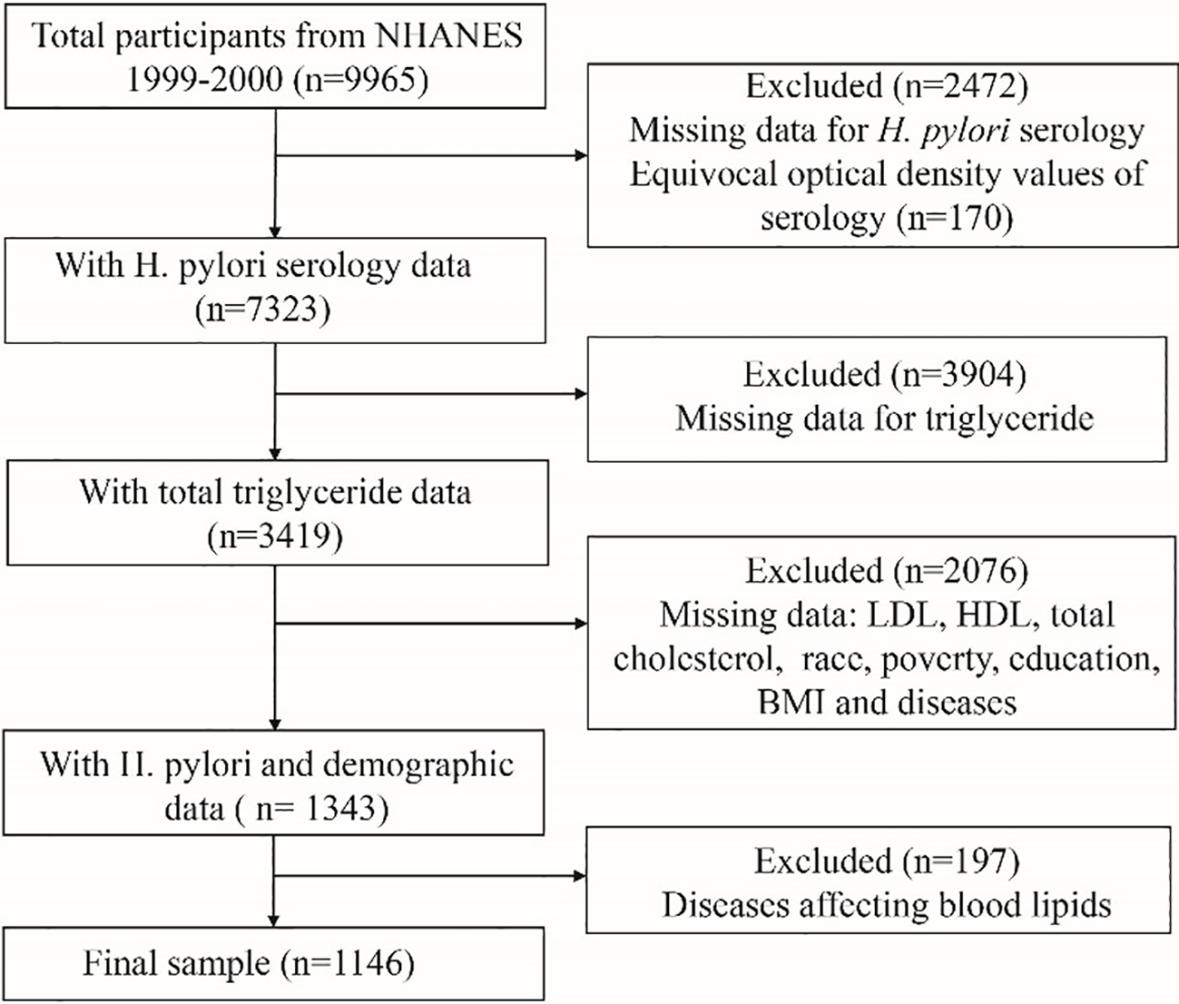
The selection flow chart of study populations.

A total of 9,965 participants were enrolled in the NHANES 1999-2000 cycle. The inclusion criteria for this study were as follows: individuals in the NHANES database who had data for serological testing for *H. pylori*, lipid laboratory testing, and demographic variables. The exclusion criteria were as follows: individuals with missing data for serological testing for *H. pylori*, lipid laboratory testing, and demographic variable information; and individuals with conditions that could potentially affect blood lipid levels, including diabetes, coronary heart disease, angina pectoris, thyroid disorders, cancer, and liver disease. A total of 1146 individuals were included in the final analysis. The flowchart of the sample selection process is shown in **Figure 1**.

### Helicobacter pylori seropositivity

As described in the NHANES protocol, serum samples were collected by venipuncture from 7,493 participants and stored at -80°C before being tested at the University of Washington. The *H. pylori* immunoglobulin G (IgG) antibodies were detected using an enzyme-linked immunosorbent assay (ELISA) kit manufactured by Wampole Laboratories (Cranbury, NJ) to determine the quantity of IgG antibodies against *H. pylori* (14). Participants were divided into two groups, *H. pylori* seropositive (optical density (OD) value ≥1.1) and seronegative (OD value <0.9), using the standard ELISA cut-off value. To avoid misleading statistical results, ambiguous values (0.9-1.1) were excluded from the analysis (15).

### Triglyceride levels

The collected samples were stored at -20°C until transported to the Lipoprotein Analysis Laboratory at Johns Hopkins University for testing. Triglyceride levels were measured enzymatically in serum or plasma using a series of coupled reactions in which triglycerides were hydrolysed to produce glycerol. Glycerol was then oxidized using glycerol oxidase, and H2O2, one of the reaction products, was converted via peroxidase to a phenazone. Absorbance was measured at 500 nm. Elevated triglyceride levels are typically defined as a fasting serum triglyceride level greater than 1.7 mmol/L (16).

### Cholesterol levels

Cholesterol is measured enzymatically in serum or plasma in a series of coupled reactions that hydrolyse cholesteryl esters and oxidize the 3-OH group of cholesterol. One of the reaction byproducts, H2O2, is measured quantitatively in a colourimetric peroxidase-catalysed reaction. Absorbance was measured at 500 nm.

### High-density lipoprotein cholesterol and low-density lipoprotein cholesterol levels

In NHANES 1999-2000, HDL-C was measured using two methods. A heparin-manganese (Mn) precipitation method and a direct immunoassay technique were used. The Heparin-Mn Precipitation Method was conducted as follows: apolipoprotein B-containing lipoproteins were removed by precipitation with heparin sulfate and MnCl2, and cholesterol was measured in the HDL-containing supernatant. The HDL-C Direct Immunoassay Method was conducted as follows: HDL was measured directly in serum. The apolipoprotein B-containing lipoproteins in the specimen were reacted with a blocking reagent that rendered them nonreactive with the enzymatic cholesterol reagent under the conditions of the assay. Absorbance was measured at 600 nm.

LDL-C was calculated from measured values of total cholesterol, triglycerides, and HDL-C according to the Friedewald calculation: [LDL-C] = [total cholesterol] – [HDL-C] – [triglycerides/5].

### Covariates

Numerous studies have shown that *H. pylori* infection is associated with various factors, such as age (17), smoking (18), alcohol consumption (19), race (17), education (20), poverty (17), and body mass index (21). In this study, covariates included age, sex, race, education level, household size, poverty-to-income ratio, BMI, alcohol consumption, smoking behaviour, and C-reactive protein. The covariates were classified as categorical variables, including sex, education level, household size, poverty-to-income ratio, BMI, alcohol consumption, and smoking behaviour, and as continuous variables, including age and C-reactive protein.

### Statistical analyses

Continuous variables are presented as the mean ± standard deviation, while categorical variables are reported as numbers and percentages. To compare baseline characteristics among different groups, the chi-square test, Student’s t test, and Fisher’s exact test were utilized as appropriate. The independent correlation between *H. pylori* seropositivity and triglyceride levels was evaluated using a logistic regression model. A P value < 0.05 was considered to indicate statistical significance. All P values were two-tailed, and the regression analysis results were presented as ORs and 95% CIs. All statistical analyses were performed using SPSS version 26.

## Results

### Clinical characteristics of study participants

**Figure 2:**
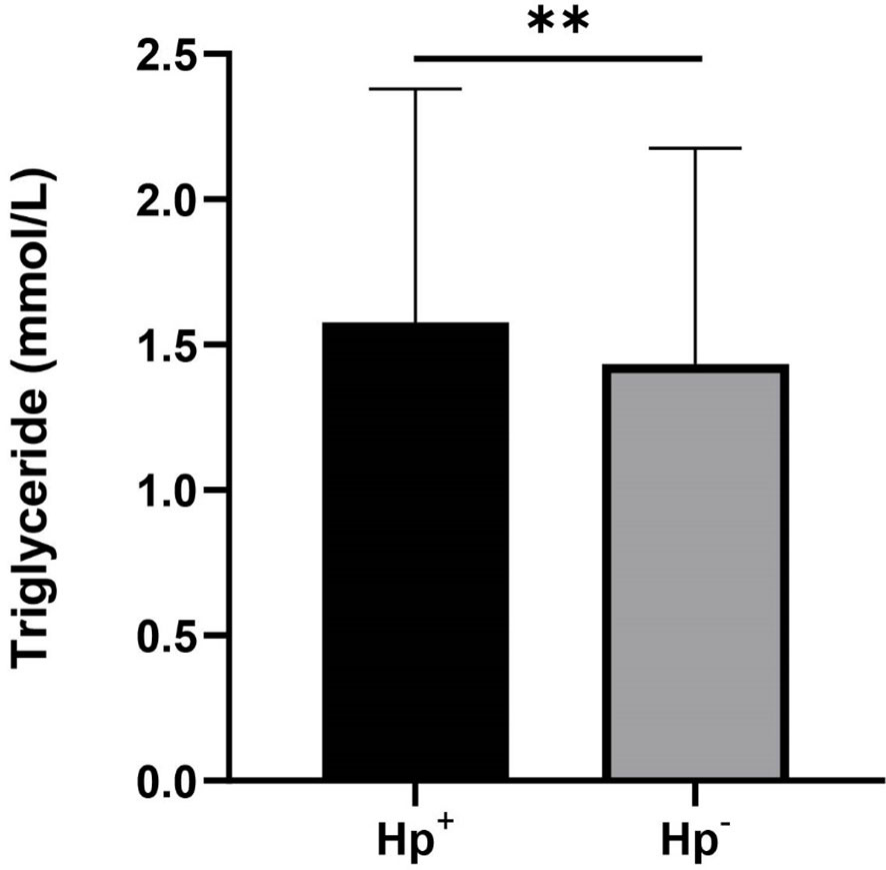
Levels of triglyceride in patients with *H. pylori* seropositive (Hp+) and *H. pylori* seronegative (Hp-). **p value < 0.01.

A total of 478 participants (41.71%) were *H. pylori* seropositive, while 668 participants (58.29%) were *H. pylori* seronegative. Significant differences were observed between the two groups in terms of age, education level, household size, and poverty income ratio (P < 0.05) **Table 1**. Notably, the *H. pylori* seropositive group had a higher mean age, lower education level, larger household size, and lower poverty income ratio compared to the seronegative group. The mean triglyceride level was also higher in the *H. pylori* seropositive group than in the seronegative group (mean triglyceride level, 2.44 mmol/L vs. 1.07 mmol/L) (**Figure 2**). However, there were no significant differences between the two groups in terms of total cholesterol, HDL-C, and LDL-C levels.

**Table 1:** Weighted characteristics of 1146 participants by H. pylori seroprevalence.

Variable	*H. pylori* seroprevalence		*P* value
	Positive(n=478)	Negative(n=668)	
Age (years)	49.45(17.47)	42.40(16.76)	**<0.001**
Gender			**0.038**
Male	245(51.30%)	301(45.10%)	
Female	233(48.70%)	367(54.90%	
Race			**<0.001**
Mexican American	203(42.50%)	102(15.20%)	
Other Hispanic	48(10.00%)	28(4.20%)	
Non-Hispanic White	104(21.80%)	406(60.80%)	
Non-Hispanic Black	109(22.80%)	110(16.50%)	
Other Race	14(2.90%)	22(3.30%)	
Educational level			**<0.001**
Less Than High School	268(56.10%)	132(19.80%)	
High School Diploma	80(16.70%)	180(26.90%)	
More Than High School	130(27.20%)	356(53.30%)	
Household size			**<0.001**
Small (1-3members)	272(56.90%)	447(66.90%)	
Large (4 or more members)	206(43.10%)	221(33.10%)	
Poverty income ratio			**<0.001**
<2	254(53.20%)	222(33.30%)	
2–4	143(29.90%)	202(30.20%)	
>4	81(16.90%)	244(36.50%)	
BMI (kg/m2)			**0.025**
Undernutrition (<18)	6(1.30%)	8(1.20%)	
Normal (18-25)	139(29.10%)	241(36.10%)	
Overweight (25-30)	183(38.20%)	203(30.40%)	
Obese (30+)	150(31.40%)	216(32.30%)	
Smoking behavior			0.090
Never	102(21.30%)	109(16.40%)	
Sometimes	16(3.30%)	21(3.10%)	
Everyday	360(75.40%)	538(80.50%)	
Alcohol behavior			0.074
Yes	311(65.10%)	468(70.10%)	
No	167(34.90%)	200(29.90%)	
CRP (mg/dL)	0.49(0.67)	0.45(0.81)	0.781
Triglyceride (mmol/L)			**0.004**
Normal (<1.7)	308(64.40%)	483(72.30%)	
Abnormal (≥1.7)	170(35.60%)	185(27.70%)	
Total cholesterol(mmol/L)	5.29(1.05)	5.24(1.07)	0.986
HDL cholesterol (mmol/L)	1.29(0.39)	1.34(0.37)	0.764
LDL cholesterol (mmol/L)	3.28(0.90)	3.24(0.93)	0.739

The meaning of the bold values is statistically significant.

### Association between Helicobacter pylori seropositivity and triglyceride levels

The results of the logistic regression analysis for the composite outcome are shown in **Table 2**. The results of univariate analysis showed eight *H. pylori* seropositivity-related variables, including age, sex, race, educational level, household size, PIR, BMI and triglycerides. Multivariate regression analysis was performed with factors showing a significant difference (P<0.05) in univariate analysis as covariates. After adjusting for confounding factors, the results showed a significant positive association between *H. pylori* seropositivity and elevated triglyceride levels (OR=1.231; 95% CI, 1.016-1.491; P=0.033). Subgroup analysis by sex revealed that there was no significant association between *H. pylori* seropositivity and triglyceride levels in males (OR=1.091; 95% CI, 0.698-1.705; P=0.704), while a significant positive association was found in females (OR=1.732; 95% CI, 1.113-2.696; P=0.015). Details are presented in **Table 3**.

**Table 2:** Univariate and multivariate logistic regression analyses of risk factors of Helicobacter pylori seropositive patients.

Variable	Univariate analysis			Multivariate analysis		
	OR	95% CI	*P* value	OR	95% CI	*P* value
Age (years)	1.024	1.017-1.031	**<0.001**	1.032	1.022-1.041	**<0.001**
Gender	0.780	0.616-0.987	**0.039**	0.869	0.657-1.149	0.324
Race	–	–	**<0.001**	–	–	**<0.001**
Educational level	–	–	**<0.001**	–	–	**<0.001**
Household size	1.532	1.202-1.952	**0.001**	1.366	0.998-1.870	0.052
Poverty income ratio	–	–	**<0.001**	–	–	0.219
BMI (kg/m2)	–	–	**0.025**	–	–	**0.036**
Smoking behavior	–	–	0.091	–	–	–
Alcohol behavior	1.257	0.978-1.614	0.074	–	–	–
CRP (mg/dL)	1.071	0.917-1.251	0.387	–	–	–
Triglyceride (mmol/L)	1.441	1.119-1.855	**0.005**	1.231	1.016-1.491	**0.033**
Total cholesterol(mmol/L)	1.048	0.938-1.171	0.987	–	–	–
HDL cholesterol (mmol/L)	0.732	0.534-1.002	0.765	–	–	–
LDL cholesterol (mmol/L)	1.038	0.913-1.179	0.740	–	–	–

The meaning of the bold values is statistically significant.

**Table 3:** Association of triglyceride level with Helicobacter pylori seropositivity based on subgroup of sex.

	Total (n=1146)			Male (n=546)			Female (n=600)		
	OR	95% CI	*P* value	OR	95% CI	*P* value	OR	95% CI	*P* value
Triglyceride	1.231	1.016-1.491	**0.033**	1.091	0.698- 1.705	0.704	1.732	1.113- 2.696	**0.015**

The meaning of the bold values is statistically significant.

## Discussion

In this study, we conducted an observational association analysis between triglyceride levels and *H. pylori* infection using the NHANES 1999-2000 dataset. To our knowledge, this study was the first to provide evidence of a significant correlation between high triglyceride levels and *H. pylori* infection in women using NHANES data. NHANES is characterized by its rigorous sampling design of the U.S. national population, high-quality research measurements, and detailed quality control procedures (15).

Our research findings indicate a positive correlation between *H. pylori* seropositivity and triglyceride levels, while no significant correlations were observed with total cholesterol, LDL-C, or HDL-C. In the stratified analysis, adjusted triglyceride levels showed a statistically significant difference with *H. pylori* seropositivity in women but not in men.

Cardiovascular disease (CVD) is a leading cause of death and disability worldwide, significantly impacting people’s lives (22). Various risk factors, such as hypertension, obesity, lack of physical activity, and diabetes, are commonly associated with CVD. Previous studies have shown that CVD is associated with several infectious pathogens, such as Chlamydia pneumoniae, cytomegalovirus, and herpes simplex virus (23). Epidemiological studies based on bacterial discovery in the past two decades have suggested a potential link between *H. pylori* infection and the incidence of heart disease (24–27), with the hypothesis that bacteria may be one of the underlying mechanisms directly or indirectly influencing heart disease (28). Inflammation or immune response caused by *H. pylori* infection in gastric mucosa is considered a major potential cause of CVD. *H. pylori* infection induces acute and chronic inflammatory responses in gastric mucosa (29, 30), which are usually systemic (31) and are associated with elevated serum fibrinogen and lipid levels, as well as the release of proinflammatory mediators, all of which are well-known risk factors for CVD (25). In addition, *H. pylori* with CagA may stimulate macrophages to produce foam cells, leading to enlargement of atherosclerotic plaques and arterial dysfunction (32). *H. pylori* infection may also promote the development of diabetes (33), and diabetes accelerates the progression of atherosclerosis (34). *H. pylori*-induced abnormalities in glucose metabolism, increased inflammatory stress, and lipid abnormalities may be underlying mechanisms of CVD.

Elevated serum levels of LDL-C are a well-known risk factor for CVD, especially coronary artery disease (35). The role of hypertriglyceridaemia in cardiovascular disease, however, is not as well established. Nevertheless, as more and more research is conducted, the evidence supporting hypertriglyceridaemia as an independent risk factor for CVD is increasing. The results from numerous large observational, epidemiological, genetic, and Mendelian randomization studies support the hypothesis that elevated levels of triglycerides in the blood, whether in the fasting or nonfasting state, are associated with an increased risk of CVD (16). Triglycerides undergo hydrolysis mediated by lipoprotein lipase (LPL), producing high concentrations of lipid products such as oxidized free fatty acids, which are associated with an increased risk of atherosclerosis and CVD through various mechanisms, including the production of cytokines, fibrinogen, clotting factors, and proinflammatory interleukins and fibrinolytic impairment (36). *H. pylori* infection activates an inflammatory response involving the expression of various cytokines, including interleukins, through a large number of polymorphonuclear and mononuclear cells (37).

It is widely believed that endogenous oestrogen during reproductive years can delay the onset of CVD in women. However, epidemiological evidence suggests that the rate of increase in CVD during menopause does not differ by age (38). Women are at a more disadvantageous position than men in terms of CVD (39). In addition, women infected with *H. pylori* have a faster rate of antibody clearance than infected men (40). This may also contribute to an increased risk of CVD in women.

Our study has some limitations. The study design was cross-sectional based on NHANES, and therefore, we cannot determine a causal relationship between serum triglyceride levels and *H. pylori* seropositivity. Additionally, NHANES only provides serological data for *H. pylori* infection, which does not differentiate between past or current infection, so we cannot accurately determine whether the infection coexists with hypertriglyceridaemia. Furthermore, our study cannot elucidate the underlying mechanisms linking *H. pylori* infection in women with hypertriglyceridaemia. Finally, the data used in this study were limited to the period between 1999 and 2000, as serum triglyceride and *H. pylori* serological data were only available for these two years in NHANES. To further substantiate our conclusions, a study with a larger sample size is needed.

## Conclusion

In summary, a positive correlation between serum triglyceride levels and H. pylori infection can be observed in females. It is crucial for female patients infected with *H. pylori* to prioritize monitoring their triglyceride levels to mitigate the likelihood of developing cardiovascular ailments.

## Data availability statement

The original contributions presented in the study are included in the article/supplementary material. Further inquiries can be directed to the corresponding author.

## Ethics statement

According to local legislation and institutional requirements, ethical review and approval were not required for research involving human participants.

## Author contributions

YX designed and supervised the study. JX collected and analyzed the data, and drafted the manuscript. JW and RZ provided advice for data analysis and manuscript writing. All authors contributed to the article and approved the submitted version.

## References

[1] Varon C Azzi-Martin L Khalid S Seeneevassen L Menard A Spuul P. Helicobacters and cancer, not only gastric cancer? Semin Cancer Biol (2022) 86:1138–54. doi: 10.1016/j.semcancer.2021.08.007 34425210

[2] Marshall BJ Warren JR. Unidentified curved bacilli in the stomach of patients with gastritis and peptic ulceration. Lancet (1984) 1:1311–5. doi: 10.1016/S0140-6736(84)91816-6 6145023

[3] Suerbaum S Michetti P. Helicobacter pylori infection. N Engl J Med (2002) 347:1175–86. doi: 10.1056/NEJMra020542 12374879

[4] Papamichael KX Papaioannou G Karga H Roussos A Mantzaris GJ. Helicobacter pylori infection and endocrine disorders: is there a link? World J Gastroenterol (2009) 15:2701–7. doi: 10.3748/wjg.15.2701 PMC2695884 19522019

[5] Wang T Li X Zhang Q Ge B Zhang J Yu L. Relationship between Helicobacter pylori infection and osteoporosis: a systematic review and meta-analysis. BMJ Open (2019) 9:e027356. doi: 10.1136/bmjopen-2018-027356 PMC6597651 31248924

[6] Malaguarnera M Bella R Alagona G Ferri R Carnemolla A Pennisi G. Helicobacter pylori and Alzheimer's disease: a possible link. Eur J Intern Med (2004) 15:381–6. doi: 10.1016/j.ejim.2004.05.008 15522573

[7] Shi WJ Liu W Zhou XY Ye F Zhang GX. Associations of Helicobacter pylori infection and cytotoxin-associated gene A status with autoimmune thyroid diseases: a meta-analysis. Thyroid (2013) 23:1294–300. doi: 10.1089/thy.2012.0630 23544831

[8] Takashima T Adachi K Kawamura A Yuki M Fujishiro H Rumi MA. Cardiovascular risk factors in subjects with Helicobacter pylori infection. Helicobacter (2002) 7:86–90. doi: 10.1046/j.1083-4389.2002.00064.x 11966866

[9] Saijo Y Utsugi M Yoshioka E Horikawa N Sato T Gong Y. Relationship of Helicobacter pylori infection to arterial stiffness in Japanese subjects. Hypertens Res (2005) 28:283–92. doi: 10.1291/hypres.28.283 16138557

[10] Mendall MA Goggin PM Molineaux N Levy J Toosy T Strachan D. Relation of Helicobacter pylori infection and coronary heart disease. Br Heart J (1994) 71:437–9. doi: 10.1136/hrt.71.5.437 PMC483719 8011406

[11] Shiotani A Miyanishi T Uedo N Iishi H. Helicobacter pylori infection is associated with reduced circulating ghrelin levels independent of body mass index. Helicobacter (2005) 10:373–8. doi: 10.1111/j.1523-5378.2005.00343.x 16181346

[12] Satoh H Saijo Y Yoshioka E Tsutsui H. Helicobacter Pylori infection is a significant risk for modified lipid profile in Japanese male subjects. J Atheroscler Thromb (2010) 17:1041–8. doi: 10.5551/jat.5157 20610892

[13] Armstrong AW Mehta MD Schupp CW Gondo GC Bell SJ Griffiths C. Psoriasis prevalence in adults in the United States. JAMA Dermatol (2021) 157:940–6. doi: 10.1001/jamadermatol.2021.2007 PMC8246333 34190957

[14] Berrett AN Gale SD Erickson LD Brown BL Hedges DW. Folate and inflammatory markers moderate the association between helicobacter pylori exposure and cognitive function in US adults. Helicobacter (2016) 21:471–80. doi: 10.1111/hel.12303 26935014

[15] Huang J Liu Z Ma J Liu J Lv M Wang F. The Association between Helicobacter pylori Seropositivity and Bone Mineral Density in Adults. Mediators Inflammation (2022) 2022:2364666. doi: 10.1155/2022/2364666 PMC9001096 35418807

[16] Reiner Z. Hypertriglyceridaemia and risk of coronary artery disease. Nat Rev Cardiol (2017) 14:401–11. doi: 10.1038/nrcardio.2017.31 28300080

[17] Graham DY Malaty HM Evans DG Evans DJ Klein PD Adam E. Epidemiology of Helicobacter pylori in an asymptomatic population in the United States. Effect of age, race, and socioeconomic status. Gastroenterology (1991) 100:1495–501. doi: 10.1016/0016-5085(91)90644-Z 2019355

[18] Ogihara A Kikuchi S Hasegawa A Kurosawa M Miki K Kaneko E. Relationship between Helicobacter pylori infection and smoking and drinking habits. J Gastroenterol Hepatol (2000) 15:271–6. doi: 10.1046/j.1440-1746.2000.02077.x 10764027

[19] Zhang L Eslick GD Xia HH Wu C Phung N Talley NJ. Relationship between alcohol consumption and active Helicobacter pylori infection. Alcohol Alcohol (2010) 45:89–94. doi: 10.1093/alcalc/agp068 19808941

[20] Moreira EJ Santos RS Nassri VB Reis AT Guerra AL Alcantara AP. Risk factors for Helicobacter pylori infection in children: is education a main determinant? Epidemiol Infect (2004) 132:327–35. doi: 10.1017/S0950268803001572 PMC2870109 15061508

[21] Suki M Leibovici WY Boltin D Itskoviz D Tsadok PT COmaneshter D. Helicobacter pylori infection is positively associated with an increased BMI, irrespective of socioeconomic status and other confounders: a cohort study. Eur J Gastroenterol Hepatol (2018) 30:143–8. doi: 10.1097/MEG.0000000000001014 29120907

[22] Li Z Lin L Wu H Yan L Wang H Yang H. Global, regional, and national death, and disability-adjusted life-years (DALYs) for cardiovascular disease in 2017 and trends and risk analysis from 1990 to 2017 using the global burden of disease study and implications for prevention. Front Public Health (2021) 9:559751. doi: 10.3389/fpubh.2021.559751 34778156 PMC8589040

[23] Wright CB Gardener H Dong C Yoshita M DeCarli C Sacco RL. Infectious burden and cognitive decline in the northern manhattan study. J Am Geriatr Soc (2015) 63:1540–5. doi: 10.1111/jgs.13557 PMC4878014 26289683

[24] Folsom AR Nieto FJ Sorlie P Chambless LE Graham DY. Helicobacter pylori seropositivity and coronary heart disease incidence. Atherosclerosis Risk In Communities (ARIC) Study Investigators. Circulation (1998) 98:845–50. doi: 10.1161/01.CIR.98.9.845 9738638

[25] Patel P Mendall MA Carrington D Strachan DP Leatham E Molineaux N. Association of Helicobacter pylori and Chlamydia pneumoniae infections with coronary heart disease and cardiovascular risk factors. BMJ (1995) 311:711–4. doi: 10.1136/bmj.311.7007.711 PMC2550716 7549683

[26] McDonagh TA Woodward M Morrison CE McMurray JJ Tunstall-Pedoe H Lowe GD. Helicobacter pylori infection and coronary heart disease in the North Glasgow MONICA population. Eur Heart J (1997) 18:1257–60. doi: 10.1093/oxfordjournals.eurheartj.a015436 9458417

[27] Whincup PH Mendall MA Perry IJ Strachan DP Walker M. Prospective relations between Helicobacter pylori infection, coronary heart disease, and stroke in middle aged men. Heart (1996) 75:568–72. doi: 10.1136/hrt.75.6.568 PMC484378 8697158

[28] Gasbarrini A Franceschi F Armuzzi A Ojetti V Candelli M Torre ES. Extradigestive manifestations of Helicobacter pylori gastric infection. Gut (1999) 45 Suppl 1:I9–I12. doi: 10.1136/gut.45.2008.i9 10457029 PMC1766655

[29] Tamer GS Tengiz I Ercan E Duman C Alioglu E Turk UO. Helicobacter pylori seropositivity in patients with acute coronary syndromes. Dig Dis Sci (2009) 54:1253–6. doi: 10.1007/s10620-008-0482-9 18770033

[30] Buzas GM. Metabolic consequences of Helicobacter pylori infection and eradication. World J Gastroenterol (2014) 20:5226–34. doi: 10.3748/wjg.v20.i18.5226 PMC4017037 24833852

[31] Yoshida N Granger DN Evans DJ Evans DG Graham DY Anderson DC. Mechanisms involved in Helicobacter pylori-induced inflammation. Gastroenterology (1993) 105:1431–40. doi: 10.1016/0016-5085(93)90148-6 7901109

[32] Santos M de Brito BB Da SF Sampaio MM Marques HS Oliveira ESN. Helicobacter pylori infection: Beyond gastric manifestations. World J Gastroenterol (2020) 26:4076–93. doi: 10.3748/wjg.v26.i28.4076 PMC7403793 32821071

[33] Marietti M Gasbarrini A Saracco G Pellicano R. Helicobacter pylori infection and diabetes mellitus: the 2013 state of art. Panminerva Med (2013) 55:277–81. 24088801

[34] Poznyak A Grechko AV Poggio P Myasoedova VA Alfieri V Orekhov AN. The diabetes mellitus-atherosclerosis connection: the role of lipid and glucose metabolism and chronic inflammation. Int J Mol Sci (2020) 21(5):1835. doi: 10.3390/ijms21051835 32155866 PMC7084712

[35] Taylor F Huffman MD Macedo AF Moore TH Burke M Davey SG. Statins for the primary prevention of cardiovascular disease. Cochrane Database Syst Rev (2013) 1:CD004816. doi: 10.1001/jama.2013.281348 PMC6481400 23440795

[36] Chapman MJ Ginsberg HN Amarenco P Andreotti F Boren J Catapano AL. Triglyceride-rich lipoproteins and high-density lipoprotein cholesterol in patients at high risk of cardiovascular disease: evidence and guidance for management. Eur Heart J (2011) 32:1345–61. doi: 10.1093/eurheartj/ehr112 PMC3105250 21531743

[37] Baggiolini M Walz A Kunkel SL. Neutrophil-activating peptide-1/interleukin 8, a novel cytokine that activates neutrophils. J Clin Invest (1989) 84:1045–9. doi: 10.1172/JCI114265 PMC329758 2677047

[38] Bots SH Peters S Woodward M. Sex differences in coronary heart disease and stroke mortality: a global assessment of the effect of ageing between 1980 and 2010. BMJ Glob Health (2017) 2:e000298. doi: 10.1136/bmjgh-2017-000298 PMC5435266 28589033

[39] Woodward M. Cardiovascular disease and the female disadvantage. Int J Environ Res Public Health (2019) 16(7):1165. doi: 10.3390/ijerph16071165 30939754 PMC6479531

[40] Longo-Mbenza B Nkondi NJ Vangu ND. Prevention of the metabolic syndrome insulin resistance and the atherosclerotic diseases in Africans infected by Helicobacter pylori infection and treated by antibiotics. Int J Cardiol (2007) 121:229–38. doi: 10.1016/j.ijcard.2006.12.003 17368586

